# Medical Association of Georgia

**Published:** 1885-05

**Authors:** 


### MEDICAL ASSOCIATION OF GEORGIA.

### Thirty-Sixth Annual Session.

### FIRST DAY—MORNING SESSION.

Savannah, Ga., April 15, 1885.

The Medical Association of Georgia convened this day at 11 o'clock in Metropolitan Hall, and was called to order by the retiring President, Dr. A. W. Calhoun, of Atlanta.

The exercises were opened with prayer by Rev. J. E. L. Holmes, Pastor of the Baptist Church.

Dr. W. H. Elliott, President of the Georgia Medical Society, in behalf of the Society, and Hon. Rufus E. Lester, Mayor of the city, in behalf of the citizens, made addresses of welcome.

Dr. T. M. Holmes, of Rome, responded in behalf of the Association.

Dr. Wm. Duncan, Chairman of Committee of Arrangements, then made his report. The following members were present:

E. K. Bozeman, Friendship; G. W. Mulligan, Washington; E. H. Richardson, Cedartown; Howard J. Williams, Macon; R. H. Hightower, Dublin; Robt. H. Taylor, Griffin; Chas. Hicks, Dublin; H. McHatton, Macon; L. G. Hardman, Harmony Grove; Jno. J. Hill, Washington; Willis B. Parks, Atlanta; D. E. Gay, Millen; Jno. D. Martin, Savannah; Geo. C. Hummel, Savannah; J. Weichselbaum, Savannah; Raymond B. Harris, Savannah; Robt. G. Norton, Effingham; T. J. Kelly, Tennille; W. P. Ponder, Forsyth; A. A. Smith, Hawkinsville; Mark H. O'Daniel, Milledgeville; T. M. Holmes, Rome; W. B. Cheatham, Dawson; J. M. Kelly, Louisville; J. R. Henderson, Sun Hill; W. H. Elliott, Savannah; A. W. Calhoun, Atlanta; E. C. Goodrich, Augusta; James A. Gray, Atlanta; J. Emmett Blackshear, Macon; T. Frank Walker, Cochran; Eugene Foster, Augusta; A. G. Whitehead, Waynesboro; Jno. A. Callaway, Milledgeville; Benj. F. Sheftall, Savannah; Jas. C. Solomon, Perry; Robt. B. Barron, Clinton; J. F. Fielder, Eastman; John B. Roberts, Sandersville; T. O. Powell, Milledgeville; E. W. Lane, Scarboro; J. W. Flanders, Wrightsville; C. H. Colding, Savannah; K. P. Moore, Macon; Wm. F. Holt, Macon; T. M. McIntosh, Thomasville; R. J. Nunn, Savannah; T. J. Charlton, Savannah; D. C. Gay, Millen; K. A. Quarterman, Walthourville; Geo. H. Stone, Savannah; J. C. LeHardy, Savannah; J. D. Wright, Louisville.

Dr. A. W. Calhoun, the retiring President, then introduced the President-elect, Dr. Eugene Foster, of Augusta, who proceeded to deliver the Annual Address. [This address will appear in the June number of THE JOURNAL.]

By Dr. T. F. Walker:

*Resolved*, That visiting physicians, the clergy, representatives of the press, and the Mayor and Council be invited to seats on the floor. Adopted.

By Dr. A. W. Calhoun:

*Resolved*, That the thanks of the Association be returned to Capt. Purse for the invitation to visit Tybee. Adopted.

The Secretary read letters from Drs. H. F. Campbell, K. P. Moore and J. McF. Gaston, expressing regrets at not being present.

The Secretary informed the President that the entire Board of Censors was absent.

The President then appointed Drs. R. J. Nunn, J. C. LeHardy, L. B. Alexander, J. Emmett Blackshear and G. W. Mulligan as Censors.

The Secretary read applications for membership from Drs. W. B. Cheatham, Dawson; R. B. Harris, Savannah; W. P. Ponder, Forsyth; J. M. Kelly, Louisville; E. W. Lane, Scarboro; Benj. S. Purse, Savannah.

On motion, they were referred to the Board of Censors.

Reports of special committees being in order, the report of the Committee on Annual Dues was called.

The Chairman of the Committee being absent, Dr. R. J. Nunn asked for further time to report. Granted.

Dr. James A. Gray stated that the Chairman of the Committees on Expert Testimony and Anatomical Material was not present, but had requested him to state that a bill had been drawn and presented to the Legislature, and that the committees to whom they were referred had reported favorably on them, and he had reasons to believe that the bills as prepared would become law at the approaching session of the Legislature.

The following report of the Committee on Publication was read and referred to the Auditing Committee, consisting of Drs. T. J. Charlton, E. K. Bozeman and S. B. Hawkins:

REPORT OF COMMITTEE ON PUBLICATION.

*To the President and Members of the Medical Association of Georgia:*

GENTLEMEN—I must again call your attention to the importance of making some provisions for having the Transactions published. It will be remembered that it was with difficulty, for lack of funds, that the volume for 1883 was published. The present volume is much larger than the previous one, and cost considerably more, and yet the same difficulties had to be contended with.

It was impossible to publish the present volume in the time prescribed by the By-laws for want of funds. I would not contract for its publication until I had secured advertisements enough to reduce the cost to a sum that the Association would be willing and able to pay. Consequently, the volume was delayed for several months, and the committee censured by individual members of the Association for this delay. Three hundred and fifty copies of the Transactions were printed at a cost of $599.64. The actual cost of the publication was reduced by $213.00 received from advertisements, making the cost per volume to the Association $1.10. Copies of the Transactions have been sent to each member of the Association, to the Secretaries of the several State

Medical Societies, to a number of the most prominent Medical Journals, to libraries, to each advertiser, and a few copies sold to individuals.

The Constitution and By-laws do not appear in this volume. It was thought best to leave them out in order to lessen the cost of the publication.

The Transactions of most of the State Medical Societies have been received by the Secretary and are at your disposal.

I have collected as follows:

| | |
|---|---|
| Cash from advertisements | $213 00 |
| Cash from Treasurer | 238 00 |
| Cash from dues | 15 00 |
| Cash from sale of Transactions | 7 00 |
| Total | $473 00 |

The following amounts have been expended:

| | |
|---|---|
| Cash paid stamps, postal cards, etc.. | $ 28 50 |
| Cash paid express on Transactions. | 44 65 |
| Cash paid postage on Transactions | 10 20 |
| Cash paid bank for collecting drafts | 2 35 |
| Cash paid telegram | 50 |
| Cash paid Jas. P. Harrison & Co. | 386 80 |
| Total | $473 00 |

JAMES A. GRAY, *Chairman.*

The resignations of Drs. W. G. Owen and C. A. Simpson were read and accepted.

The Board of Censors reported favorably on the applications for membership of Drs. W. B. Cheatham, R. B. Harris, W. P. Ponder, J. M. Kelly, E. W. Lane and Benj. S. Purse. Adopted.

The President stated that he thought it well to consider some better plan for publishing the annual Transactions, and suggested that it would be well to have a committee to examine into the matter and report at this meeting.

By Dr. A. W. Calhoun:

*Resolved*, That a committee of one from each Congressional district be appointed to consider this question of having the Transactions published in journal form. Adopted.

The President appointed the following on that committee: Drs.

T. J. Charlton, W. B. Parks, S. B. Hawkins, W. B. Cheatham, L. B. Alexander, T. M. Holmes, J. J. Hill, L. G. Hardman, E. C. Goodrich.

Dr. R. J. Nunn asked that a special hour this afternoon be given to reading a paper by Dr. Geo. C. Hummel on The Methods of Microscopical Examinations, and the Artificial Culture of Bacteria.

On motion, 4 o'clock was named as the hour.

On motion of Dr. J. C. LeHardy, the Association adjourned to 3:30 o'clock p. m.

### FIRST DAY—AFTERNOON SESSION.

Association called to order by Dr. Eugene Foster, President.

At 4 o'clock Dr. Geo. C. Hummel read paper already reported by title, and demonstrated the artificial culture of bacteria.

By Dr. J. Emmett Blackshear:

*Resolved*, That the thanks of the Association be, and are hereby tendered Dr. Hummel for the able paper just read. Adopted.

The following report of the Committee on Prize Essays was read and adopted:

*To the President of the Medical Association of Georgia:*

The Committee on Prize Essays begs leave to report: At the last meeting the fund on hand was $79.18, including interest to that date. The committee was then authorized to put the fund at interest, which has been done, and the committee has now on hand $84.75.

No essays have been presented for competition, but this inures to the benefit of the fund, which, by accruing interest, will ultimately become sufficiently valuable to attract attention. The money is in the hands of the chairman of the committee, subject to the orders of the Association. Your committee would recommend that the fund be invested in a bond, and that the annual statement inviting competition be continued

R. J. NUNN, M. D.,
*Ch'n on behalf of Committee.*

*Savannah, Ga., April 15, 1885.*

On motion, the report was received and adopted.

The report of Sections being in order, the President requested the Secretary to call the Sections.

FIRST DISTRICT.—Practice, no report ; Surgery, report by W. H. Elliott ; Gynecology, report by Dr. R. J. Nunn.

SECOND DISTRICT.—No report.

THIRD DISTRICT.—Practice, report by Dr. A. A. Smith ; Surgery, no report ; Gynecology, report by Dr. T. F. Walker.

FOURTH DISTRICT.—No report.

FIFTH DISTRICT.—No report on Practice and Gynecology ; report on Surgery by Dr. R. H. Taylor.

SIXTH DISTRICT.—Practice, report by Dr. W. O'Daniel; Surgery and Gynecology, no report.

SEVENTH DISTRICT.—No report on Practice and Gynecology ; Surgery, report by Dr. E. H. Richardson.

EIGHTH DISTRICT.—Dr. J. J. Hill made report on Practice ;no report on Surgery and Gynecology.

NINTH DISTRICT.—No report.

TENTH DISTRICT.—No report on Practice and Gynecology; Dr. T. R. Wright made a report on Surgery.

Upon a call for voluntary contributions to be read by title, and called up and read at the pleasure of the Association, the following were presented:

"*Trachomatous Tumors within the Larynx in Children—Removal under Influence of twelve per cent. Solution of Cocaine*," by A. W. Calhoun, M. D., Atlanta.

"*Aphasia in Brain Disease*," by Mark H. O'Daniel, M. D., Milledgeville.

"*A Case of Tuberculosis supposed to have Originated from Arsenious Acid Poisoning*," by T. M. Holmes, M. D., Rome.

"*Tabes Mesenterica*," by Charles Hicks, M. D., Dublin.

"*Hemorrhagic Malarial Fever*."—A. G. Whitehead, M. D., Waynesboro.

"*Syphilis—The importance of its thorough diagnosis and long continued treatment*," by J. C. LeHardy, M. D., Savannah.

"*Glaucoma following the excessive use of Atropia*," by J. M. Hull, Augusta.

"*The best nutriment for the bony system*," by R. J. Nunn, M. D., Savannah.

"*A new method of treating diphtheria*," by R. J. Nunn, M. D., Savannah.

"*Some of the physiological and therapeutical properties of alcohol*," by J. P. Stevens, M. D., Macon.

"*A case of double uterus with fœtus in each*," by E. W. Lane, Scarboro.

"*Concentric enlargement of the wrist in hereditary syphilis*," by R. O. Engram, Montezuma.

"*Notes on Hyperidrosis*," by Henry Wile, M. D., Philadelphia. Presented by James A. Gray, M. D., Atlanta.

"*A successful method of using jaborandi in acute and chronic Bright's disease*," by F. H. O'Brien, M. D., Atlanta.

"*Contraction of the sphincter ani muscle*," by W. F. Westmoreland, M. D., Atlanta.

Dr. Mark H. O'Daniel stated that the name of Dr. I. L. Harris, of Milledgeville, had been dropped from the roll, and that the Doctor desired to be reinstated.

On motion, Dr. Harris was restored by paying the dues for this year.

On motion, Dr. W. H. Elliott, of Savannah, read report already presented by title.

The Association then adjourned to meet at 8 o'clock this evening to hear the address of the orator.

### FIRST DAY—EVENING SESSION.

The Association was called to order at 8 o'clock by the President, Dr. Eugene Foster, who introduced the orator, Dr. Howard J. Williams.

Dr. Williams then delivered the annual oration, after which the Association adjourned to meet at 10 o'clock Thursday morning.

---

### SECOND DAY—MORNING SESSION.

The Association was called to order by the President at 10 o'clock.

The minutes of the previous day were read and approved.

By Dr. E. H. Richardson :

*Resolved*, That, in view of the very great importance to the public of the matters discussed in the President's address, it be placed at the disposal of the secular press. Adopted.

Secretary read telegrams from Drs. W. F. Westmoreland, J. B. S. Holmes and J. T. Johnson, expressing regret at being absent.

By Dr. J. B. Roberts:

*Resolved*, That the Association, appreciating the love and remembrance of Dr. J. T. Johnson, now of California, return the following by telegraph : "Association returns greeting with hope of your success." Adopted.

The application of Dr. R. A. Quarterman, of Walthourville, was read and referred to the Board of Censors, who reported favorably, and he was elected a member of the Association.

Dr. T. M. Holmes, of Rome, read paper already reported by title.

On motion, the name of Dr. T. R. Wright, of Augusta, was added to the Committee on Anatomical Material.

At 11 o'clock, the Association adjourned, and went on an excursion to Tybee.

### EVENING SESSION.

Association called to order at 8 o'clock by the President.

The Nominating Committee was announced as follows : A. G. Whitehead, Burke county; S. B. Hawkins, Sumter county; K. A. Quarterman, Liberty county; R. G. Norton, Effingham county; W. F. Holt, Bibb county; E. W. Lane, Emanuel county; T. M. McIntosh, Thomas county; J. C. Solomon, Houston county; J. J. Hill, Wilkes county; T. M. Holmes, Floyd county; A. A. Smith, Pulaski county; T. J. Kelly, Washington county; W. B. Cheatham, Terrell county; J. C. LeHardy, Chatham county; R. H. Taylor, Spalding county; R. B. Barron, Jones county; M. H. O'Daniel, Baldwin county; J. W. Flanders, Johnson county.

The President appointed the following delegates to the American Medical Association: S. B. Hawkins, temporary chairman, Americus; A. G. Whitehead, Waynesboro; W. F. Westmoreland, Atlanta; T. O. Powell, Milledgeville; A. W. Calhoun, Atlanta; M. G. Hatch, Tennille; J. C. LeHardy, Savannah; Robert Battey, Rome; R. J. Nunn, Savannah; DeSaussure Ford Augusta; B. R. Doster, Blakely; J. B. Roberts, Sandersville; James A. Gray, Atlanta; C. H. Hall, Macon; C. D. Smith, Newnan; C. L. Sample, Cannoochee; John Gerdine, Athens; G. W. Mulligan, Washington; Wm. O'Daniel, Bullards; W. D. Bizzell, Atlanta; J. W. Flanders, Wrightsville; E. H. Richardson, Cedartown; K. P. Moore, Macon; E. L. Conolly, Atlanta; H. F. Campbell, Augusta; L. B. Alexander, Forsyth; J. Weichselbaum, Savannah; T. F. Walker, Cochran; W. H. Elliott, Savannah; L. G. Hardman, Harmony Grove; Wm. Duncan, Savannah; James A. Fort, Americus; J. M. Hull, Augusta; T. M. Macintosh, Thomasville; W. F. Holt, Macon; A. A. Smith, Hawkinsville; H. H. Steiner, Augusta; J. C. Solomon, Perry; B. F. Sheftall, Savannah; E. C. Goodrich, Augusta; J. McF. Gaston, Atlanta.

On motion, the President's name was added to the list.

On motion, Dr. E. H. Richardson, of Cedartown, read report on Surgery for the Seventh Congressional district.

On motion, Dr. R. J. Nunn read paper on Diphtheria, reported by title yesterday.

Association adjourned to 9:30 o'clock to-morrow.

THIRD DAY—MORNING SESSION.

The Association called to order by the President at 9:30 o'clock.

The minutes of the previous day were read and approved.

The Auditing Committee made the following report, which was adopted:

SAVANNAH, April 16, 1885.

The committee appointed to examine the accounts of the Secretary and Treasurer find them correct, and recommend that Dr. J. M. Hull's expenses, as acting treasurer at Macon, be paid by the Association.

T. J. CHARLTON,
E .K. BOZEMAN,
S. B. HAWKINS,
*Committee.*

The special committee appointed to consider the matter of publishing the Transactions in journal form made the following report, which was adopted :

The committee recommend that the Association accept the proposition of the ATLANTA MEDICAL AND SURGICAL JOURNAL, viz.: The Atlanta Journal proposes to publish the Transactions in the JOURNAL for one year, furnish each member with the JOURNAL and pay all expenses of the Secretary and Treasurer's offices (except their expenses to and from the meetings) for $2.00 per member.

We also recommend that the members shall not receive the JOURNAL until their dues are paid in full.

Signed:

T. J. CHARLTON,
L. B. ALEXANDER,
T. M. HOLMES,
W. B. CHEATHAM.
W. B. PARKS,
L. G. HARDMAN,
J. J. HILL,
E. C. GOODRICH.

The Nominating Committee made the following report, which was adopted :

*President*—R. J. Nunn, Savannah.

*First Vice-President*—L. B. Alexander, Forsyth.

*Second Vice-President*—T. F. Walker, Cochran.

*Censor*—E. H. Richardson, Cedartown.

*Committee on Publication*—James A. Gray, E. C. Goodrich, Robt. Battey, H. V. M. Miller, W. D. Bizzell, J. P. Logan.

*Committee on Necrology*—K. P. Moore, A. G. Whitehead, G. W. Holmes, J. S. Todd.

*Committee on Prize Essays*—R. J. Nunn, Robt. Battey, W. A. Love, H. F. Campbell, Eugene Foster.

*Committee of Arrangements*—E. C. Goodrich, J. M. Hull, C. W. Hickman, Eugene Foster, W. H. Doughty, jr., T. R. Wight.

SECTIONS—FIRST DISTRICT.

*Practice*—W. Duncan, K. A. Quarterman, J. J. Waring.

*Surgery*—W. H. Elliott, R. G. Norton, G. H. Stone.
*Gynecology*—R. B. Harris, E. W. Lane, J. Weichselbaum.

SECOND DISTRICT.

*Practice*—T. A. Chappell, W. B. Cheatham, L. B. Bouchelle.
*Surgery*—T. M. McIntosh, W. S. Wood, J. G. Hopkins.
*Gynecology*—A. P. Taylor, B. R. Doster, P. S. Hilsman.

THIRD DISTRICT.

*Practice*—A. A. Smith, Chas. Hicks, E. K. Bozeman.
*Surgery*—S. B. Hawkins, J. D. Herman, A. K. Taylor.
*Gynecology*—T. F. Walker, J. F. Fielder, R. O. Ingram.

FOURTH DISTRICT.

*Practice.*—C. D. Smith, W. A. Pool, W. W. Fitts.
*Surgery.*—J. T. Slaughter, J. A. Beasly, J. F. Cole.
*Gynecology.*—A. W. Griggs, Geo. B. Grimes, Paul Faver.

FIFTH DISTRICT.

*Practice*—W. S. Elkin, W. H. Pool, Henry Gaither.
*Surgery*—James A. Gray, W. B. Parks, R. H. Taylor.
*Gynecology.*—W. A. Love, J. McF. Gaston, T. S. Powell.

SIXTH DISTRICT.

*Practice*—C. H. Hall, J. C. Solomon, W. O'Daniel.
*Surgery*—Lewis Harris, H. McHatton, A. J. Williams.
*Gynecology*—K. P. Moore, T. O. Powell, L. B. Alexander.

SEVENTH DISTRICT.

*Practice*—T. M. Holmes, W. S. Kendrick, F. R. Calhoun.
*Surgery*—W. B. Wells, J. B. S. Holmes, E. H. Richardson.
*Gynecology*—Robert Battey, J. W. Clements, C. P. Gordon.

EIGHTH DISTRICT.

*Practice*—J. J. Hill, A. A. Bell, J. C. Pope.
*Surgery*—S. C. Bennedict, E. D. Alfriend, R. B. Nisbet.
*Gynecology*—John Gerdine, A. Moody Burt, G. W. Mulligan.

NINTH DISTRICT.

*Practice*—J. B. Pendergrass, J. H. Goss.
*Surgery*—L. G. Hardman, W. J. Bush, R. L. Harris.
*Gynecology*—J. W. Bailey, W. A. Watson.

TENTH DISTRICT.

*Practice*—S. D. Brantley, A. H. Baker, J. M. Kelly.

*Surgery*—DeSaussure Ford, T. R. Wright, M. G. Hatch.

*Gynecology*—H. F. Campbell, W. H. Doughty, A. G. Whitehead.

By Dr. Wm. F. Holt:

*Resolved*, That the Association decline with thanks the invitation to visit Montgomery. Adopted.

By Dr. Wm. F. Holt:

*Resolved*, That the President appoint a committee of seven to inquire into the advisability of permanently locating the Association in one of the larger cities of the State, and that the committee report at the next meeting of the Association. Adopted.

The following compose the committee: Drs. Wm. F. Holt, A. G. Whitehead, Robert Battey, H. V. M. Miller, R. J. Nunn, G. J. Grimes, Eugene Foster.

By Dr. R. J. Nunn:

*Resolved*, That the Secretary be requested to inquire into the cost of printing reprints, and to contract for the reprints at as low a figure as the work can be done. Adopted.

A resolution from Dr. Nunn changing the name of the Committee on Anatomical Material to that of Medical Legislation, and requesting the committee to take such action as is necessary to have the government admit free of duty all medicines and surgical supplies, was adopted.

Drs. Lane, McIntosh and Soloman each read papers of great interest to the profession, and brought out general comment.

Capt. D. G. Purse, having been sent for, appeared in the hall, and was escorted to a seat at the side of the President. Dr. Blackshear, of Macon, in a happy manner, presented Captain Purse, in behalf of the Association, with an elegant gold-headed cane as a testimonial of its appreciation of the royal manner in which he entertained its members.

Capt. Purse, in reply, returned his most hearty thanks, and assured the gentlemen that it was the proudest day of his life to be thus honored by such an intelligent and refined body as the Medical Association of Georgia.

The paper of Dr. Whitehead on cases of Hemorrhagic Malarial Fever produced considerable discussion. Dr. Whitehead took the ground that quinine did very little, if any, good in cases of this kind. He relied mainly on calomel and chinoidine, and always pushed the calomel to tyalism, after which he used chlorate potash and muriate tincture of iron. He regards tyalism as important. He gives the calomel in two-grain doses every hour until specific effect is obtained.

Dr. Wm. F. Holt asked if inunction of the mercury would not produce the desired effect. Dr. Whitehead thought it might, but he had never used it in that manner.

Dr. McHatton said: Calomel may be curative in these cases, but it certainly is not a prophylactic in all of them. The last patient that I saw had taken thirty grains in the previous thirty-six hours. I was called to see him and found him in his initial chill. I look upon hæmaturia as a misnomer. From my limited experience with the disease, I look upon it as a hæmoglobinuria, the chemical constituents of the blood being the principal, if not the only important characteristic of the urine. In many cases I failed entirely to find any blood corpuscles, and when they were found they bore no relation in number to the size and number of hæmin crystals that could be produced from the liquid. Councelman and Abbott have found a hyaline body in the red blood corpuscle in profound malarial poisoning. Michifara and Celli describe a microbe attacking directly the red blood corpuscle in malarial diseases.

My opinion is that the damage is done in the circulation at large, and that a greater or less number of red blood corpuscles are destroyed, thus giving us their chemical constituents in the urine. This pathological condition of the blood occurs in other diseases, namely—in poisoning by chlorate of potash, phosphorous and arsenic; also, in typhus and scorbutus. If the etiology of this disease is malarial, it seems to me that quinine must be our sheet-anchor.

This paper was also discussed by Drs. LeHardy, McIntosh and Alexander.

On motion, Dr. Calhoun gave a synopsis of the paper already reported by title.

By Dr. R. G. Norton:

*Resolved*, That the thanks of the Association are due the citizens of Savannah, the press of the city, and the several railroads in the State, for courtesies extended the Association. Adopted.

Augusta was selected as the next place of meeting.

By Dr. James A. Gray:

*Resolved*, That when the Association adjourn to-day it adjourn to meet in Augusta on the third Wednesday in April, 1886. Adopted.

The President, in a happy manner, thanked the members for their uniform kindness to him, and adjourned the Association.

James A. Gray, M. D., *Secretary*.

