# Correction

**Published:** 1885-06

**Authors:** 


A CORRECTION.—In the minutes of the Association, as published in the last issue of THE JOURNAL, there appeared no report from the ninth district. There was a report on gynecology, made by Dr. L. G. Hardman, of Harmony Grove, and it should have so read.

